# Pre-clinical xenotransplantation: physiology and pharmacy in human decedent and non-human primate models

**DOI:** 10.3389/frtra.2025.1576549

**Published:** 2025-04-17

**Authors:** Douglas J. Anderson, Jayme E. Locke

**Affiliations:** ^1^ Department of Surgery, University of Alabama at Birmingham, Birmingham, AL, United States; ^2^ NYU Langone Health, New York, NY, United States

**Keywords:** xenotransplant, kidney, renal physiology, pharmacokinetics, non-human primate, decedent model

## Abstract

Non-human primates and decedent humans have emerged as the two principal translational models in xenotransplantation. Each model has differing advantages and drawbacks. In this manuscript, we will compare and contrast the relative strengths of each model, focusing on the physiologic function of the xenograft in a human decedent or non-human primate. Additionally, we will discuss the pharmacologic agents typically employed in each model, highlighting both the ability of the decedent model to test clinically-relevant medication strategies that may be impossible in non-human primates due to species-specificity.

## 1 Introduction

For most patients with end-stage organ disease, transplantation represents the best available treatment. Unfortunately, the shortage of available organs for transplant results in many patients never reaching transplantation and dying from their organ failure. In the case of end-stage kidney disease, dialysis may offer a life-preserving alternative but still falls well short of the outcomes achieved by kidney transplantation (1). In the United States, the insufficient supply of donor kidneys has resulted in an ever-lengthening waiting list, with average waiting times exceeding 4 years (2). In other forms of organ failure, an alternative therapy akin to dialysis is less readily available, and many patients will die while awaiting an organ that never arrives.

Xenotransplantation represents the most readily available solution to this donor organ shortage. While artificial or bioengineered organs offer many theoretical advantages, the realization of these technologies is likely many years away, whereas the recent developments in laboratory and preclinical research into xenotransplantation have progressed to the point of clinical translation. Indeed, 6 human recipients of porcine xenografts (2 heart, 4 kidney) have been reported (3–7).

The experience gained from these 5 recipients will undoubtedly be critical in the further development of the field. However, there is still much that can be learned in pre-clinical translational models that will help understand the immune response to a xenograft and examine the function of that xenograft post-transplantation. These experiments may help improve the outcomes for xenotransplant recipients, broaden the scale of xenotransplant availability, or help develop the next generation of genetically-modified source animals. In this manuscript, we will review the two major pre-clinical models in kidney xenotransplantation: non-human primates (NHP) and human decedents. We will compare and contrast these models, focusing on their ability to examine the physiology and pharmacology of kidney xenotransplantation.

## 2 Pre-clinical models in xenotransplantation

### 2.1 Donor source animals

Early attempts at xenotransplantation favored NHP as the source of donor organs owing to the genetic similarity to humans. However, in the modern era, pigs have emerged as the preferred source animal. Increasing understanding of the immunology of xenotransplant over several decades, along with the introduction of CRISPR-Cas9 gene editing, has led to the creation of donor animals with multiple genetic modifications. While numerous differing genetic constructs have been developed and tested, there are several similarities across virtually all pigs used in pre-clinical testing. First, knockout of at least one of the three major carbohydrate xenoantigens: αGal, N-glycolylneuraminic acid (Neu5Gc), and the Sd(a) blood group antigen (8–10). Second, insertion of one or more human transgenes selected to abrogate the immune response to the graft (11–16). A list of human transgenes which have been inserted is included in Table 1. Third, additional genetic modifications chosen for practical reasons have sometimes been studied, such as knockout of the growth hormone receptor to slow organ growth or knockout of the porcine endogenous retrovirus (PERV) genome to reduce the risk of zoonosis (16, 17).

**Table 1:** Human transgenes employed in xenotransplantation.

Gene	Target product	Purpose of modification
Complement regulators		
*CD46*	Membrane Cofactor Protein (MCP/CD46)	Inhibits complement by binding C3b/C4b
*CD55*	Complement decay-accelerating factor (DAF/CD55)	Indirectly blocks formation of membrane attack complex
*CD59*	MAC-inhibitory protein (MAC-IP/protectin/CD59)	Blocks the membrane attack complex by inhibiting C9 polymerization
Anti-inflammatory genes		
*HMOX1*	Heme oxygenase 1 (HO-1)	Inhibits production of inflammatory mediators
*TNFAIP3*	TNF-α induced protein 3 (A20)	Reduces ischemia-reperfusion injury, inflammation and apoptosis
*CD47*	Integrin associated protein (IAP/CD47)	Suppresses macrophage response via binding with SIRP-α
Coagulation regulators		
*THBD*	Thrombomodulin (TM)	Reduces platelet aggregation
*PROCR*	Endothelial protein C receptor (EPCR/CD201)	Enhances activation of Protein C

### 2.2 Non-human primate model

Non-human primates are the predominant model in pre-clinical transplantation research. Compared with the inbred rodent models favored for mechanistic studies, outbred NHPs offer a more dynamic and human-like immunologic environment to test immunosuppression strategies or xenografts. The phylogenetic proximity of NHPs to humans means many of the medications used will be effective in either species and the native renal physiology is similar between humans and NHPs. The most commonly used species are Rhesus macaques (Macaca mulatta, in allotransplantation) or baboon (Papio papio, in xenotransplantation), though occasionally other species such as cynomolgus macaques (Macaca fascicularis) are considered (18). These NHP species are significantly smaller than humans, typically between 5 and 15 kg when used in transplant experiments. As such, the porcine xenografts must be recovered from younger donor animals, before they have grown prohibitively large. This potentially increases the risk of technical complications when dealing with small vasculature.

Kidney xenotransplant experiments using the NHP model typically involve removal of both native kidneys, leaving the animal entirely dependent on the implanted xenograft. The animals are treated with the desired immunosuppression and monitored closely for urine output, laboratory values, and any signs of rejection or other complication. Post-transplant follow-up can extend out indefinitely, and experiments lasting more than 2 years have been reported (16).

### 2.3 Human decedent model

As the design of the genetically modified donor pig became more complex, and aimed for future human recipients, cross-species differences between humans and NHPs became increasingly apparent. For example, *in vitro* studies suggested knockout of all 3 carbohydrate antigens would reduce reactivity and improve crossmatch results in human recipients. However, these triple knockout cells had more reactive crossmatches with NHP sera than cells with only two of the antigens removed (19). Additionally, complex biologic medications such as antibodies or fusion proteins may have a degree of species specificity and may not be testable in NHPs.

These identified gaps between NHPs and humans led to the development of the decedent model. In these experiments, a recently-deceased, brain-dead human, deemed ineligible or unsuitable for organ donation, serves as the transplant recipient. Again, the native kidneys may be removed to better understand the function of the xenograft. Various immunosuppression strategies can be attempted, without concern for differing species-specific effects. The decedent does require continuous ICU-level support to overcome the pathophysiology of brain death, and this also limits the duration of follow-up. Furthermore, the rarity of the decedent and these experiments necessitates optimization of each individual study, which introduces additional variability and reduces reproducibility. Like clinical transplantation, no two decedent studies are exactly alike.

## 3 Assessment of xenograft physiology

### 3.1 Kidney xenograft function

In both models, the simplest, and likely most crucial, assessment is the measurement of renal graft function and glomerular filtration rate (GFR). In both models, serial creatinine levels can demonstrate trends in function. When both native kidneys have been removed, this function can be entirely attributed to the graft. Other measures of graft function such as cystatin C levels or inulin clearance studies can be obtained.

In NHPs, serum creatinine is similar to or lower than typically seen in humans, owing to the animals' smaller size. The earliest reports of functioning xenotransplants in non-human primates come from the mid 1990s, but in these cases, graft function was short lived (20). Through significant effort, graft survival gradually improved (20, 21). More recently, graft survival with normal serum creatinine over 2 years after xenotransplantation has been demonstrated. In these cases, serum creatinine has remained within the normal range (between 1 and 2 mg/dl) (16, 22). Translation of serum creatinine to GFR is possible using equations similar to pediatric human GFR equations (23), though direct measurement through clearance studies remains the gold standard.

The two earliest reports of xenotransplantation in the decedent model failed to show definitive creatinine clearance (24, 25). In both cases the xenografts produced urine, but in one case the creatinine did not fall while in the other, the native kidneys remained in place which prevented full attribution of the improving creatinine to the xenograft. Subsequent studies have convincingly shown creatinine clearance in the decedent model (26). In one study, GFR was measured to exceed 200 ml/min by both creatinine clearance and inulin clearance approaches (27).

### 3.2 Renin-angiotensin-aldosterone system (RAAS)

**Figure 1:**
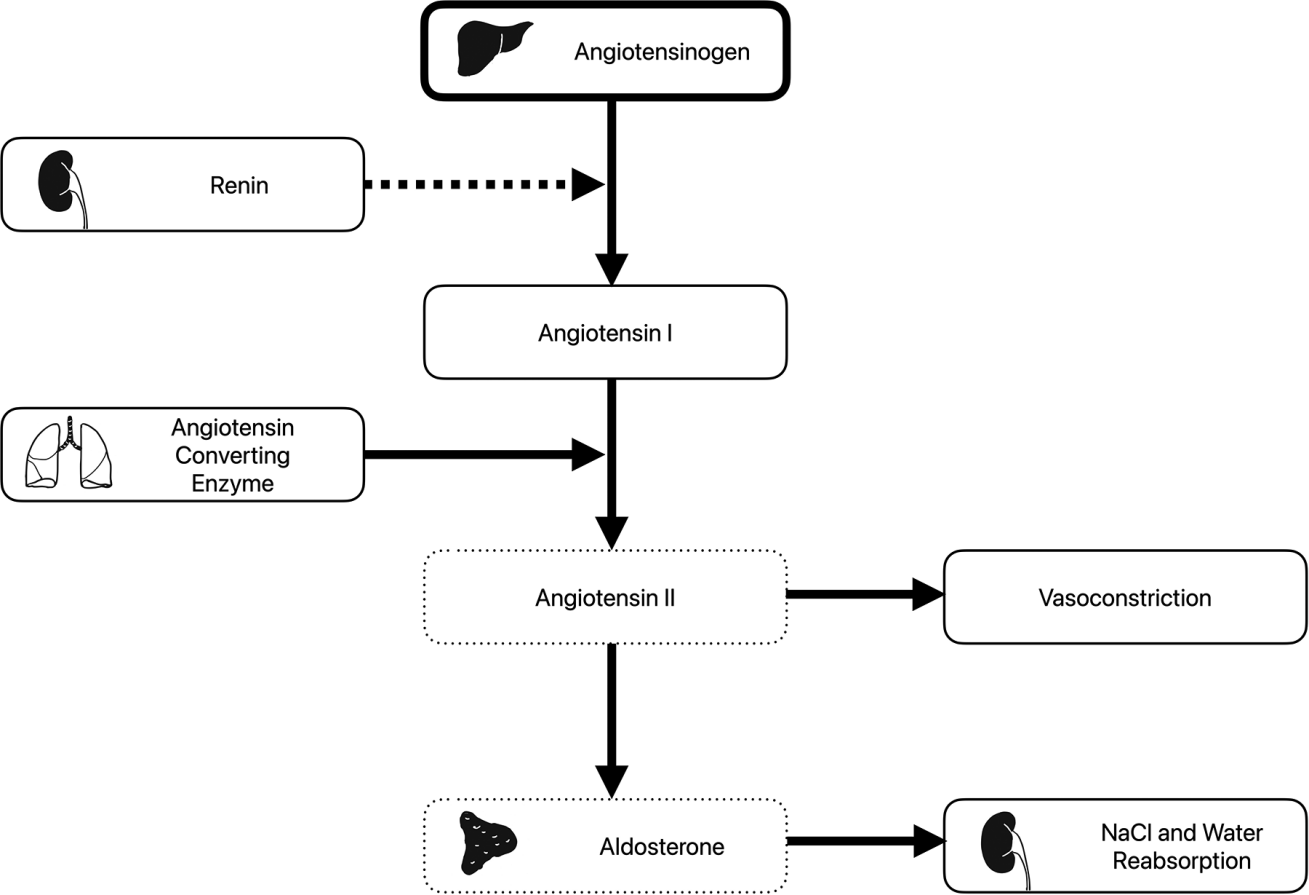
The renin-angiotensin-aldosterone system. Angiotensinogen produced in the lung is converted to angiotensin I by renin produced by the kidney. Angiotensin converting enzyme from the lung then converts angiotensin I to angiotensin II. This has direct vasoconstricting effect but also leads to release of aldosterone from the adrenal gland, which acts to increase salt and water reabsorption in the kidneys. Following xenotransplantation, elevated angiotensinogen levels (thick box) and decreased angiotensin II and aldosterone levels (dotted boxes) have been observed. Of note, while plasma renin levels were normal, plasma renin activity was low (dashed arrow).

Previous concerns have been raised about reduced efficacy of porcine renin to cleave human or primate angiotensin (Figure 1). In a pig-to-baboon xenotransplantation study, the baboons were shown to have similar renin and angiotensin II level post-xenotransplant, despite increased angiotensinogen levels (28). Renin was produced over the 5 month follow-up period at relatively stable levels. Despite the production of renin, measured renin activity levels were low. Angiotensinogen levels increased in the serum and urine over the follow-up, suggesting inefficient conversion to angiotensin I. However, angiotensin II and aldosterone levels remained fairly constant, and similar to pretransplant levels. This implies enough angiotensin I was being produced to meet physiologic demands. The authors hypothesized the porcine renin had reduced, but not absent, activity. Furthermore, they suggested this was likely not to be prohibitive given preserved blood pressure and electrolyte levels.

In a similar study in the decedent setting (27), reduced levels of plasma renin activity, angiotensin II, and aldosterone were seen, despite normal plasma renin and angiotensin I levels. Over the course of the follow-up period, angiotensin II levels increased from 0.6 to 10.6 pg/ml. Again, the clinical relevance of this was unclear, as serum potassium and magnesium levels were maintained with supplementation and the decedent remained hemodynamically stable. This suggests at least some residual RAAS activity, which could be entirely attributed to porcine renin.

Unlike the experimental settings described above, in the case of clinical xenotransplantation, it is likely one or both native kidneys will remain in place and may continue to produce human renin. However, this may be highly variable between individuals depending on the underlying cause and duration or renal disease. Regardless, the stability of blood pressure and electrolyte balance in both NHP and decedent models supports that the porcine xeno-kidney and porcine renin will be able to provide adequate RAAS activity to maintain physiologic homeostasis.

### 3.3 Parathyroid hormone and calcium metabolism

Phosphorus and calcium levels are typically higher in pigs than in primates, raising concern about the metabolism of these electrolytes or suppression of parathyroid hormone (PTH) production post-xenotransplantation (29, 30). Experiments in non-human primates have shown phosphorus levels remaining in the lower end of the normal range for primates, while calcium levels approached those seen in pigs. Observations have been made suggesting this may suppress PTH production or secretion (29).

Contrary to the NHP results, in the decedent model, PTH has been observed to spike following xenotransplantation (27). This was an appropriate response to low ionized calcium levels which required IV supplementation. PTH levels decreased over the follow-up period, again following the normalizing ionized calcium levels on the second day post-operatively. Serum phosphate levels remained elevated in this study, despite normal urinary phosphate excretion. These data suggest intact PTH signaling to the porcine xenograft.

### 3.4 Water handling and volume

Compared with primates, the porcine kidney has fewer long-loop nephrons and a reduced ability to concentrate urine (31). Additional species-specific differences between primate and porcine vasopressin may further reduce urine concentration (27). A syndrome of clinical hypovolemia and hypotension seen in NHP xenotransplant recipients (32) may be partially attributed to this reduced concentrating power. Several studies have observed this phenomena, which is typically managed with IV fluid supplementation. Measurement of urine osmolality post-transplant in NHP showed decreased osmolality (<300 mOsm), which would be inappropriate in the setting of hypovolemia, further reinforcing the idea that the porcine kidney was not as able to concentrate the urine (28).

In the decedent model, the pathophysiology of brain-death confounds vasopressin metabolism. Exogenous vasopressin is typically administered, as hypothalamic production ceases with brain death. In the decedent recipient, extreme quantities of urine were observed over the first 24 h, resulting in significant hypernatremia, and consistent with the NHP experience (27). Urine osmolality increased from 230 mOsm/kg to 436 mOsm/kg from post-transplant day 1–6, though this may have been falsely elevated due to the presence of significant glucosuria. Urine output and serum sodium normalized over the follow-up period. Despite concerns about differences between porcine and human vasopressin, evidence of intact human vasopressin signaling on aquaporin channels in the collecting ducts of the xenograft has been shown.

### 3.5 Erythropoeitin

Under normal conditions, the kidney produces erythropoietin in response to hypoxia and anemia, promoting the production of additional red blood cells in the bone marrow. In NHP xenotransplant recipients, gradual development of anemia has been observed. However, whether this is attributable to differing triggers for erythropoietin production in the pig kidney, reduced response in the NHP host, or due to an unrelated cause or stressor is unknown (29). To date, there have been no assessments of erythropoietin production or effect in the decedent models, owing to the short follow-up period. However, this is unlikely to be a barrier to clinical translation as exogenous erythropoietin can be administered, just as it is for many dialysis patients.

## 4 Pharmacology of xenotransplantation

### 4.1 Immunosuppressive medications

As noted above, NHPs are the preferred pre-clinical model for testing immunosuppression strategies in both allo- and xenotransplantation. The NHP immune system is far more dynamic than that of laboratory-bred rodents, and the similarities to human immune system allow for cross reactivity to a large number of medications. However, responses in NHP are not always predictive of responses in humans. Life-threatening cytokine storm seen in human subjects receiving TGN1412 had not been observed in cynomolgus monkeys, and the pro-thrombotic effect of the anti-CD40l hu5c8 was only seen in rhesus monkeys, but not cynomolgus monkeys (33). Furthermore, as high affinity biologic agents are developed, species specificity potentially becomes a barrier, as different medications would be required for a NHP or human recipient. For example, rabbit anti-thymocyte globulin (rATG) is generated specifically against human thymocytes. In non-human primates, significantly higher doses (up to 4-fold) are required to achieve sufficient lymphocyte depletion (34). Additionally, eculizumab, which is a C5 inhibitor frequently used in transplantation for treatment of antibody-mediated rejection and atypical hemolytic-uremic syndrome, is species-specific (35), and is ineffective in non-human primates.

A wide variety of immunosuppressive approaches have been tested in the NHP model. Depletional induction is utilized in the virtually all xenotransplant protocols. Conventional immunosuppression with calcineurin inhibition has typically been insufficient to prevent rejection of the xenograft in NHP (36–38), leading to adoption of costimulatory blockade-based approaches (22, 37). Inhibition of the CD40-CD154 pathway has been found to be particularly effective, especially when targeting CD154 directly. All long-term xenograft survival observed in non-human primates has been in the setting of CD154 blockade. No CD154 blockade agents are clinically available for use in humans at present, however the clinical heart and kidney xenotransplant recipients have received an investigational CD154 blockade medication, tegoprubart, based on the importance of this pathway in the NHP model (4, 39). Contrary to the NHP experience, the decedent model has been utilized to show conventional immunosuppression is capable of preventing rejection of a 10-gene edit porcine xenograft (24, 25), suggesting clinical translation may not require the additional of experimental immunosuppressive medications.

Experiments in both NHP and decedent models have demonstrated the importance of complement in both hyperacute and acute humoral rejection. In the NHP model, persistent xeno-antibody to triple-knockout pigs has been observed. Therefore, complement inhibition has been used to reduce the risk of hyperacute rejection (37). Even with insertion of complement mediating human genes, complement deposition has been observed following xenotransplantation (24, 40). NHP studies have used a variety of complement inhibitors, including cobra venom factor (37, 41), C1 inhibitor (42), and C5 inhibitor (36). In the decedent model, complement deposition with early thrombotic microangiopathy has been observed (40). Subsequent studies have shown the species-specific C5 inhibitor, eculizumab, prevents complement deposition and no thrombotic microangiopathy was seen.

### 4.2 Pharmacokinetics

It is also worth considering whether a porcine xenograft will demonstrate similar pharmacokinetics and drug metabolism to a human kidney. While immunosuppressive drug levels have been monitored for dose adjustments in NHP models, there have been no direct studies of the pharmacokinetics of medications in NHP xenograft recipients. In the decedent model, studies assessing the pharmacokinetics of common immunosuppressive and antibiotic medications have occurred. Calculated half-lives and AUC for tacrolimus and mycophenolate mofetil were similar to reference ranges when dosed consistent with clinical practice (27, 43). Furthermore, observed vancomycin pharmacokinetics in a decedent xenotransplant recipient closely followed those predicted by a pooled population model (43). This suggests that medication pharmacokinetics are likely to be similar between a human kidney and xenograft, and large alterations in dosing regimens are not likely to be required.

## 5 Discussion

Over the past several years, there has been remarkable progress towards the clinical translation of xenotransplantation. Multiple genetic edits have lowered the immunologic barriers which once seemed insurmountable. Despite these advances, there are still knowledge gaps which will need to be addressed prior to widespread adoption of xenotransplantation. The two models described here have helped narrow many of these gaps. To continue the progress in xenotransplantation, each model should be understood and appreciated for its relative strengths and weaknesses.

The pig-to-NHP model of xenotransplantation has long been the gold-standard pre-clinical model. It offers the opportunity to test the genetic modifications of the donor pig or immunosuppression strategies in a near-human immune environment, and then follow that response over a relatively long period of time. The physiology of NHPs is sufficiently similar to humans in most respects. Experiments in NHP have been able to predict adequate xenograft function to prevent uremia and normal electrolyte homeostasis. Furthermore, issues with urine concentration and water handling in NHP experiments were subsequently confirmed in human decedents. Importantly, NHP experiments are more easily repeated, albeit in modest numbers. However, as donor pigs have been increasingly optimized for human recipients and advanced biologic therapies are developed, species specificity is becoming an increasing limitation of the NHP model.

In contrast, the decedent model offers the opportunity to test the donor organs and medications in a fully human environment, without risk to a living human. However, this approach has two major drawbacks. The pathophysiology of brain death and the practicalities of supporting the decedent mandate a relatively short follow up period, typically no more than a week or two. Significant exogenous support has been required, including vasopressin and vasopressors. Indeed, significant physiologic derangements, such as multisystem organ failure and disseminated intravascular coagulation, have occurred, clouding the interpretation of results (24). One case has been followed out to 61 days (44), but this should likely be considered an exception rather than the norm. Regardless, it is still significantly shorter than the multi-year follow-up possible in NHPs. Second, suitable decedent recipients are relatively rare, as they need to be sufficiently stable to allow for the experiment to proceed while simultaneously being unsuitable for organ donation. From an ethical perspective, every opportunity for human organ donation must be exhausted before a decedent can be considered for participation in xenotransplant experiments. This, paired with the diversity of medical histories and clinical scenarios surrounding each decedent renders each experiment unique. The rarity of these experiments requires that each experiment be adjusted to incorporate the lessons learned in the previous experiment. These reports should be interpreted more as a case series than a repeated experiment in the traditional sense.

As xenotransplantation continues its path towards the clinic, both models will be needed to provide critical information addressing remaining questions and concerns. Data from these types of experiments will also supplement the small, but growing experience in clinical xenotransplantation. There have now been 2 porcine heart and 4 porcine kidney transplants reported (3, 4, 39). Pairing the experience gleaned from these few cases with thoughtfully designed experiments will help expand the reach of xenotransplantation so that it can begin to help address the vast need for donor organs.
